# Flow Characteristics of the Entrance Region with Roughness Effect within Rectangular Microchannels

**DOI:** 10.3390/mi11010030

**Published:** 2019-12-25

**Authors:** Haiwang Li, Yujia Li, Binghuan Huang, Tiantong Xu

**Affiliations:** National Key Laboratory of Science and Technology on Aero-Engine Aero-thermodynamics, Beihang University, Beijing 100191, China; 09620@buaa.edu.cn (H.L.); zy1804311@buaa.edu.cn (Y.L.); Huangbh@buaa.edu.cn (B.H.)

**Keywords:** entrance length, microchannels, aspect ratios, roughness, flow characteristics

## Abstract

We conducted systematic numerical investigations of the flow characteristics within the entrance region of rectangular microchannels. The effects of the geometrical aspect ratio and roughness on entrance lengths were analyzed. The incompressible laminar Navier–Stokes equations were solved using finite volume method (FVM). In the simulation, hydraulic diameters ($D_{h}$) ranging from 50 to 200 µm were studied, and aspect ratios of 1, 1.25, 1.5, 1.75, and 2 were considered as well. The working fluid was set as water, and the Reynolds number ranged from 0.5 to 100. The results showed a good agreement with the conducted experiment. Correlations are proposed to predict the entrance lengths of microchannels with respect to different aspect ratios. Compared with other correlations, these new correlations are more reliable because a more practical inlet condition was considered in our investigations. Instead of considering the influence of the width and height of the microchannels, in our investigation we proved that the critical role is played by the aspect ratio, representing the combination of the aforementioned parameters. Furthermore, the existence of rough elements obviously shortens the entrance region, and this effect became more pronounced with increasing relative roughness and Reynolds number. A similar effect could be seen by shortening the roughness spacing. An asymmetric distribution of rough elements decreased the entrance length compared with a symmetric distribution, which can be extrapolated to other irregularly distributed forms.

## 1. Introduction

Due to the rapid development of micro-electro-mechanical systems (MEMS), the flow mechanisms in microchannels have been a hot research topic worldwide [1,2,3,4,5,6]. At the same time, microfluidics are increasingly applied in aero engines, gas turbines, electrical devices, and so on. It has been observed that the conventional formulations applicable to macro-size structures may be invalid for micro-structures [1,2,3,4,5,6,7,8,9]. Therefore, research investigating the flow behavior in microchannels is in great demand.

In contrast to macroscale flows, the majority of microscale flows are laminar, and the relatively small dimensions of microchannels may cause a high pressure drop. The aspect ratio of the microchannel plays an important role due to the relatively small size. Chen et al. [10] studied the effect of the aspect ratio on laminar flow bifurcations in curved rectangular tubes driven by pressure gradients, and derived the ranges of stable flow solutions. In their further study [11], they investigated the effects of the aspect ratio on multiple flow solutions in a two-sided parallel motion lid-driven cavity. They distinguished the regions of stable and unstable flows according to the different aspect ratios. Besides, unlike in conventional devices, in many micro-size channels, the length of the channel is always not sufficient to give rise to fully developed flows [12]. Hence, it is of great significance to estimate the entrance length of microchannels, especially under relatively lower Reynolds numbers.

The entrance region can be defined as the area from the inlet of the channel to a location where the maximum local velocity has gained 99% of its fully developed value [10]. Since the fluid needs a much longer distance to shape into a fully developed flow pattern, this criterion is applicable in engineering for estimation of the length of the entrance region. It is important to estimate the entrance length in microchannels because the transport properties are highly dependent on this region. Besides, considering the relatively short dimension of the channel length, the entrance effects of micro-size flow should be paid more attention. 

Previous investigations of the entrance length have mainly focused on the Reynolds number and the geometric parameters of the channels such as the hydraulic diameter and aspect ratio. The entrance length in conventional channels has been studied by many scholars since the 1960s. Atkinson et al. [13] and Chen et al. [14] conducted a numerical investigation to estimate the effect of the Reynolds number on the entrance region in macroscale circular pipes and between two parallel plates. Atkinson et al. [13] found that the dimensionless entrance region length was linearly related to the Reynolds number as in Equation (1); meanwhile, Chen et al. [14] proposed the correlation shown in Equation (2), where $L_{e}$ is the length of the entrance region and $D_{h}$ is the hydraulic diameter of the channel. Coefficients $C_{1}$ and $C_{2}$ are listed in Table 1. Schlichting et al. [15] considered boundary theory and proposed that the dimensionless entrance region length in macro-size devices was directly proportional to the Reynolds number as shown in Equation (3). Muzychka and Yovanovich et al. [16] simplified the mathematical models and then proposed new models for square channels which showed a good agreement with correlation proposed by Schlichting et al. [15].

(1) $$\frac{L_{e}}{D_{h}}=C_{1}+C_{2}Re$$

(2) $$\frac{L_{e}}{D_{h}}=\frac{C_{1}}{C_{2}Re+1}+C_{3}Re$$

(3) $$\frac{L_{e}}{D_{h}}=C_{1}Re$$

However, the behaviors of entrance length in macrochannels and microchannels are obviously different. 

The effect of viscous force plays a bigger role in microscale flow than at the conventional scale [17,18], arousing controversy as to whether the classical fluid theory is applicable to microscale fluids. Hence, it is important to estimate the entrance length in microscale flow. However, unlike conventional macroscale channels, the applicable results of entrance lengths in microscale devices are limited.

Micro-PIV (particle image velocimetry) experiments carried out by Lee and Kim et al. [19,20] using deionized water flowing about *Re* = 1 in a rectangular channel with 58 μm depth, 100 μm width, and 30 mm length showed that the entrance lengths of the microscale channels were much shorter than those of macroscale channels. Ahmad and Hassan et al. [21] conducted experimental investigations to estimate the entrance lengths of rectangular microchannels with micro-PIV. They proposed new correlations of Reynolds numbers and entrance lengths. Hao et al. [22] analyzed the development process of laminar flow in trapezoidal microchannels. The hydraulic diameter was 273 μm, and micro-PIV was adopted. They found that the correlation between entrance length ($L_{e}$) and the Reynolds number (Re) was $L_{e}/D_{h}=0.08-0.09Re$.

Renksizbulut and Niazmand et al. [23] carried out simulations to study the laminar flow as well as heat transfer in the entrance region of the trapezoidal models. The aspect ratio ranged from 0.5 to 2 and the Reynolds numbers from 10 to 1000. The results showed that the previous studies that calculated the entrance lengths based on fully developed flow were inaccurate, and the entrance length was a function of Reynolds number and geometric parameters. They proposed new correlations to estimate the length of the entrance region. However, the inflow condition they used in their simulation was a uniform velocity profile, which is different from the realistic conditions in the inlet of a channel. 

Galivis and Yarusevych et al. [24] numerically studied the entrance region length of microchannels with hydraulic diameters between 100 and 500 µm. The Reynolds numbers ranged from 0.5 to 200 and aspect ratios from 1 to 5. The authors concluded that when the Reynolds number (*Re*) was under 50, the dimensionless entrance lengths changed nonlinearly with Reynolds numbers but this correlation became linear for higher Reynolds numbers. Besides, the dimensionless entrance length increased with increasing channel aspect ratios under a certain *Re*. However, as in the research of Renksizbulut and Niazmand et al. [23], the velocity in the entrance was given as a uniform profile, which does not represent real inlet conditions.

Although some studies on the entrance length in microscale channels have been done, as Table 2 presents, the previous studies were mainly qualitative, and the applicable results are still limited—especially for laminar flow at low Reynolds numbers. Even though some of them proposed correlations of entrance length [23,24], the inlet conditions were given as a uniform velocity in these studies, which does not simulate real inlet velocities. Therefore, further study is necessary to explore the factors influencing the entrance length in microscale channels. This paper conducted simulations to estimate the factors influencing the dimensionless entrance region length. In addition, we aimed to quantitatively research the entrance lengths in different channels. Compared with giving a uniform velocity profile at the entrance as in previous studies, we used a new inlet configuration to simulate a realistic inlet velocity profile in this research, making the results more reliable.

## 2. Method of Investigation 

### 2.1. Physical Model and Computation Domain

**Figure 1:**
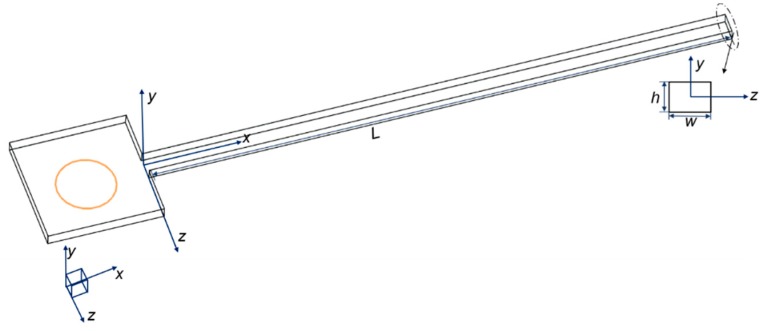
Computational domain used for simulation.

As illustrated in Figure 1, the computational domain included a giant reservoir and a long microchannel, which were connected by fillets. The presence of a giant reservoir can eliminate the fluctuation of flow in order to ensure stability before entering the channel. It can also prevent the pre-developing velocity profile from affecting the inlet of the channel. The inlet position was a hole in the middle of the reservoir. The channel was a cuboid domain with 10 mm length (*L*). Square channels with hydraulic diameters of 100, 150, and 200 μm and rectangular channels with aspect ratios (width/height) of 1, 1.25, 1.5, 1.75, and 2 were investigated. Considering the criterion that the length and width of the reservoir should be at least 100 times the microchannel hydraulic diameter, their values were both 2 mm. 

### 2.2. Numerical Methods 

With deionized water used as the working fluid, the numerical model for fluid flow in the microchannel was developed under the following assumptions:

(1) Steady three-dimensional fluid flow.
(2) Laminar and incompressible.
(3) Continuum assumption is applicable.

Based on the above assumptions, the governing equations for conservation of mass and momentum in the microchannel can be written as given below in tensor form:

Continuity equation:

(4) $$\frac{\partial }{\partial x_{i}}\left(\rho u_{i}\right)=0$$

Momentum equation:

(5) $$\frac{\partial }{\partial x_{i}}\left(\rho_{f}u_{i}u_{j}\right)=-\frac{\partial p}{\partial x_{j}}+\frac{\partial }{\partial x_{i}}[\mu_{f}\left(\frac{\partial u_{j}}{\partial x_{i}}+\frac{\partial u_{i}}{\partial x_{j}}\right)$$

where *ρ* is the fluid density, u is the average flow velocity, and *p* is the pressure.

The simulations were conducted for a range of Reynolds numbers from 0.5 to 100. The Reynolds number is defined as

(6) $$Re=\frac{\rho u_{m}D_{h}}{\mu }$$

where *ρ* is the fluid density, $u_{m}$ is the average flow velocity, $D_{h}$ is the hydraulic diameter of the microchannel, and *μ* represents the fluid viscosity. The hydraulic diameter is defined as $D_{h}=\frac{2wh}{\left(w+h\right)}$, with *w* being the width and *h* the height of the channel.

The hydrodynamic boundary conditions were:

(1) At the channel wall surface (no slip), *u = v = w =* 0;
(2) At the inlet, *x = −*2.02 mm, *u =* $u_{in}$
(3) At the outlet, *x = L*, *p =* $p_{out}$ *=* 1 atm.

The gridding was carried out in ICEM CFD 15.0. In order to obtain high-quality grids, structured hexahedral elements were adopted with mesh refinement near the walls. With the criterion of ICME CFD that the value representing grid quality should be greater than 0.3, these values in the current research were all over 0.6, satisfying the criterion. Mesh independence studies were performed to achieve the balance between solution accuracy and computation time. The error (Δ) was defined as in Equation (7), where $\Delta p_{f}$ is the pressure drop obtained from the fine grids and $\Delta p_{c}$ represents the value gained from the coarse grids:

(7) $$\Delta =\frac{\Delta p_{f}-\Delta p_{c}}{\Delta p_{c}}$$

Table 3 shows the grid independence results in a square channel with $D_{h}$ = 100 μm and *Re* = 50. For this case, Grid4 was chosen and this work was applied for all the models with different Reynolds numbers.

### 2.3. Simulation Validation

In order to certify the accuracy of the grids and the computational methods, we conducted simulations on the previous experiments. Based on the experiments in Ahmad et al. and Li et al.’s researches. [21,25], all of the tested channels were square cross-sectional areas. Simulations were performed with square microchannels where $D_{h}=200\;\mu m$. 

**Figure 2:**
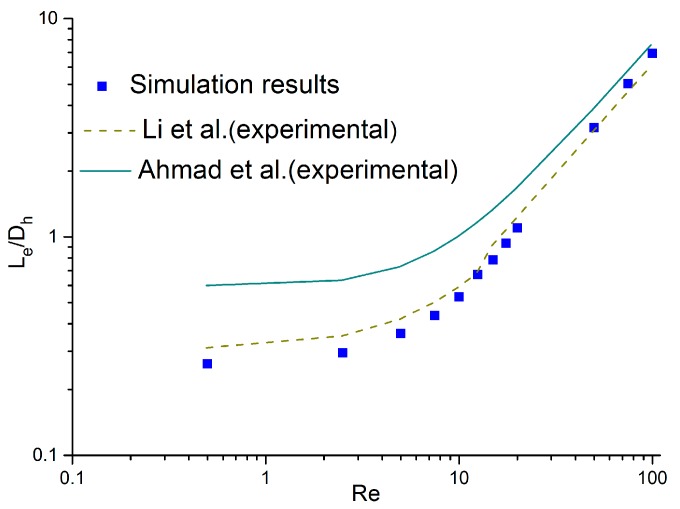
Comparison between the simulation results and the experimental data [21,25].

The comparison chart is shown in Figure 2. As Figure 2 presents, the calculation results in this paper are represented by scatter plots, and the experimental results in the references are presented in lines based on the correlations proposed in these literature. 

Note that the values of $L_{e}/D_{h}$ in Ahmad et al.’s research [21] are higher than in Li et al.’s research [25] and this work. This discrepancy mainly contributed to the distinction of inlet configuration between Ahmad et al.’s research [21] and Li et al.’s research [25]. As the authors explained in Ahmad et al.’s research [21], the inlet configuration in their experiments was asymmetrical, since the height difference between the top surfaces of the reservoir and the channel was considerable and the difference of the bottom surfaces was much smaller. As a result, the velocity profile was shifted down and the maximum velocity was not located in the measured mid-depth plane. Hence, an extra distance was necessary in their experiments to shift the maximum velocity back to the mid-depth plane and to achieve a fully developed profile. However, the experiments in Li et al.’s research [25] avoided this geometric asymmetry to ensure the maximum velocity was located in the measured plane. As shown in Figure 1, the models in this paper were also symmetric. Therefore, the values of $L_{e}/D_{h}$ in the current work are smaller than Ahmad et al.’s research [21], and the slight deviation between Li et al.’s research [25] and this work may have contributed to the uncertainty measurement of the experiments. Therefore, the simulation methods and the grid-computing methods are believable. Simulations in this paper were all conducted using the methods above.

## 3. Results and Discussion 

### 3.1. Correlations of Dimensionless Entrance Lengths 

Simulations were performed to propose the correlations of the dimensionless entrance lengths in microchannels with different aspect ratios (*w*/*h*). When the hydraulic diameter is kept constant, the shorter distance between parallel walls is narrowed with increasing aspect ratio. In order to prevent the geometry from affecting the development of the boundary layer, the aspect ratios of the microchannels in the previous studies were usually between 1 and 2 (1 ≤ w/h ≤ 2). Hence, five channels were used in this research. The hydraulic diameter kept constant at 200 µm ($D_{h}$ = 200 µm) and the aspect ratios were 1, 1.25, 1.5, 1.75, and 2, respectively, with the Reynolds numbers ranging from 0.5 to 100. The parameters of the five channels are shown in Table 4.

**Figure 3:**
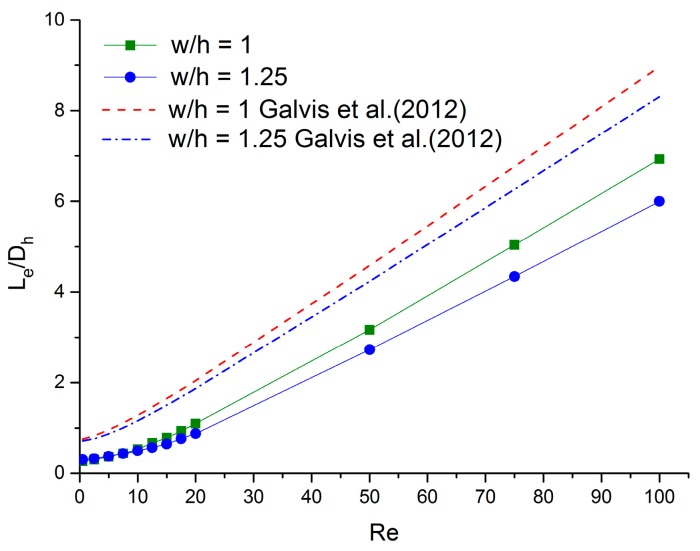
Comparison between simulation results and the previous simulation study [20].

The comparison between simulation results and the previous simulation study [24] is shown in Figure 3. There are two groups of channels with aspect ratios of 1 and 1.25. The entrance length estimated by study [24] is longer than in this work. This discrepancy may be a result of the different inlet conditions. The present study set a reservoir before the inlet of the microchannels to simulate real inlet conditions for the flow in the microchannels. Galvis et al. [24] gave a uniform velocity at the entrance. The uniform velocity may need a longer distance to become a fully developed velocity profile. Hence, the entrance lengths are longer than in this work.

**Figure 4:**
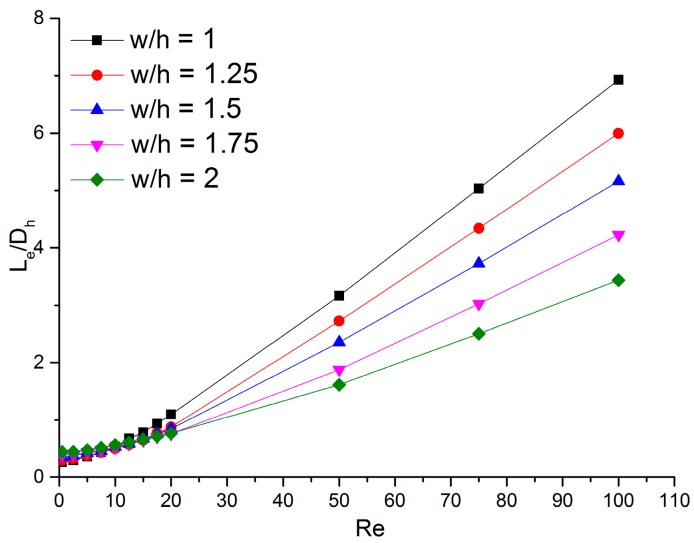
Variation of the dimensionless entrance region length with the Reynolds number at different aspect ratios.

The variations of the dimensionless entrance region length ($L_{e}/D_{h}$) with the Reynolds number (*Re*) in all five microchannels are shown in Figure 4. All these plots share a common trend: the correlation is nonlinear for *Re* ≤ 15 but it becomes linear as the Reynolds number increases. This trend is consistent with the experimental conclusion in Hao et al.’s research [22]. In addition, it also indicates that microchannel aspect ratios have no significant effect on dimensionless entrance region lengths for *Re *≤ 15, but for *Re* ≥ 15, the dimensionless entrance length decreases as the channel aspect ratio grows for a given Reynolds number. Additionally, the gap between different channels increases with increasing Reynolds number. The channels with lower aspect ratio need a longer entrance region for higher Reynolds numbers; this may contribute to the fact that the influence of the wall viscous force is stronger in microchannels with lower aspect ratios. Since a lower aspect ratio means the walls are closer to each other when the hydraulic diameters are similar. It should be noted that the calculated difference between two parts of the correlation for 12.5<Re<15 was less than 8.6%. Therefore, the Reynolds number range from 12.5 to 15 is considered as a transition region for the entrance length, and both nonlinear and linear correlations could be applied.

Based on the previous study and the simulation results, a piecewise function is adopted for all five microchannels in the form:

(8) $$\frac{L_{e}}{D_{h}}=\{\begin{array}{c} \frac{C_{1}}{C_{2}Re+1}+C_{3}Re\;(0<Re\leq 12.5)\\ C_{4}\times Re+C_{5}(15<Re\leq 100) \end{array}$$

The coefficients of the modified correlations are listed in Table 5. We concluded that the dimensionless entrance length was linearly dependent on the Reynolds number when *Re* > 15; thus, the correlations can be adopted for laminar flow with Reynolds numbers higher than 100.

The velocity profiles at the entrance (*x* = 0) of the five channels with different aspect ratios (*w*/*h*) are shown in Figure 4, and they are compared with the theoretical fully developed velocity profile, which is shown as a black solid line. In the plots, the vertical distances (*y*) are normalized with the half-height of the channel ($y_{max}$), while the axial velocities (*u*) are normalized with the theoretical maximum axial velocity ($u_{FD}$), which is considered as the fully developed velocity at the centerline.

**Figure 5:**
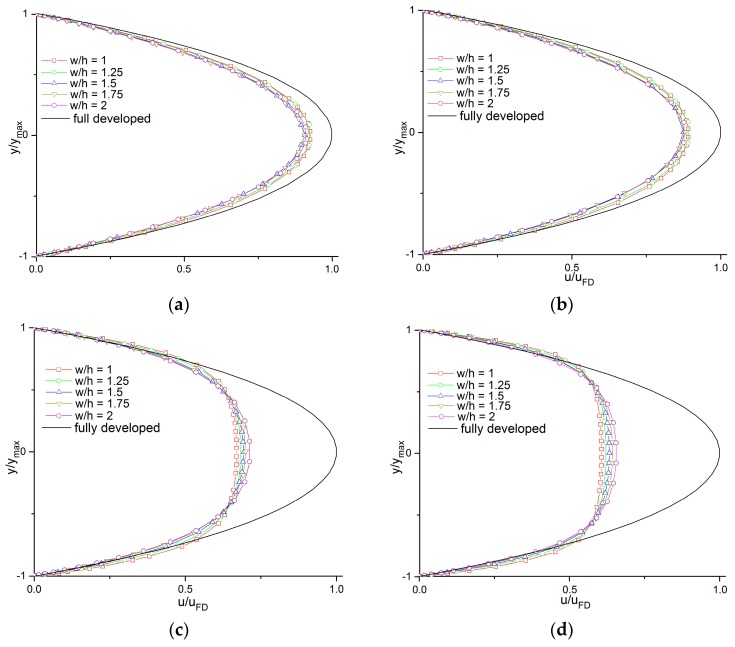
Velocity profiles at the entrance of the channel with different aspect ratios at *Re* of (**a**) 0.5, (**b**) 5, (**c**) 50, and (**d**) 100.

As Figure 5 illustrates, for lower Reynolds numbers as in Figure 5a,b, the velocity profiles at the entrance of all five microchannels were approximated as close to the fully developed velocity profiles, indicating that the aspect ratio had an insignificant influence on the entrance effect. However, with Reynolds numbers of 50 and 100 as in Figure 5c,d, the impact of the aspect ratio (*w*/*h*) on the velocity profiles at the entrance became more evident: for channels with relatively lower aspect ratios, the values of $u/u_{FD}$ were smaller and the velocity profiles were flatter. This means that for higher Reynolds numbers, the lower aspect ratio (*w*/*h*) channels needed longer entrance regions to transform their velocity profile until it was fully developed.

### 3.2. Verification of Correlations 

The correlations have been proposed to estimate the entrance lengths of microchannels with different aspect ratios. However, only one model was researched for each aspect ratio. Hence, the correlations should be verified to ensure that these correlations are applicable for other models with the same aspect ratio but different widths and heights. Two groups of models were simulated.

Group 1 were all square channels with an aspect ratio of 1 (w/h=1) and the cross-sectional areas of the three models were w×h = 100 × 100 µm^2^, 150 × 150 µm^2^, and 200 × 200 µm^2^, respectively. Group 2 were all rectangular channels with an aspect ratio of 2 (w/h=2) and the cross-sectional areas of the three models were w×h = 100 × 50 µm^2^, 200 × 100 µm^2^, and 300 × 150 µm^2^, respectively. 

From the above research, the microchannel entrance length correlations for w/h=1 and w/h=2 microchannel are as in Equations (9) and (10), respectively:

(9) $$\frac{L_{e}}{D_{h}}=\{\begin{array}{c} \frac{0.263}{0.267Re+1}+0.0471Re\;(0<Re\leq 15)\\ 0.0707\times Re-0.29\;(15<Re\leq 100) \end{array}$$

(10) $$\frac{L_{e}}{D_{h}}=\{\begin{array}{c} \frac{0.421}{0.061Re+1}+0.0265Re\;(0<Re\leq 15)\\ 0.0302\times Re+0.1631\;(15<Re\leq 100) \end{array}$$

**Figure 6:**
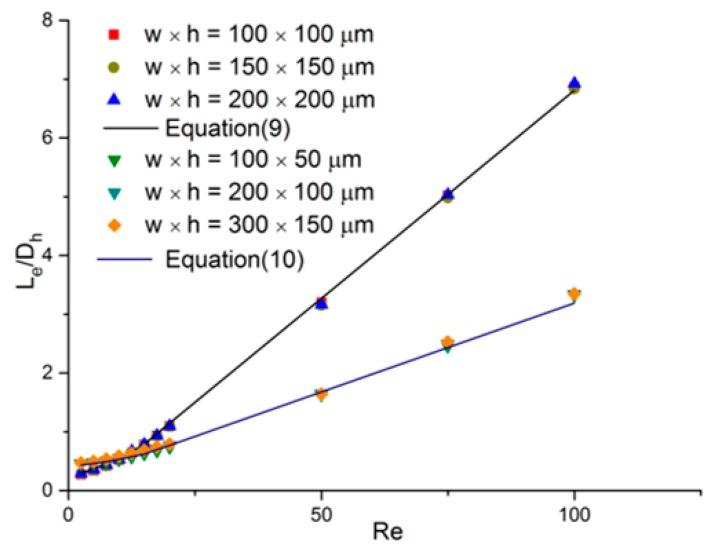
Dimensionless entrance region length comparison between new correlation and simulation results in microchannels with different widths and heights.

Figure 6 shows the variation of the dimensionless entrance region length ($L_{e}$/$D_{h}$) with the Reynolds number (*Re*) using the simulation results obtained in this study. The simulation results are shown by scatter plots, while Equations (9) and (10) are shown as solid lines in the graph at the same time. 

The following can be observed from the graph: (1) The difference in the values of $L_{e}$/$D_{h}$ in each group of channels for a given Reynolds number was less than 3%; (2) The deviation between the correlations and the simulation results were all within 4%. These can be attributed to the fact that the dimensionless entrance region length was constant for a certain Reynolds number in microchannels with the same aspect ratio regardless of the widths, heights, and hydraulic diameters. Hence, it can be said that Equation (9) is applicable for all microchannels with an aspect ratio of 1, and Equation (10) can be applied to all microchannels with an aspect ratio of 2. The same generalization can be extended to the other formulas in Section 3.1 with different aspect ratios. This promotes the universality of the correlations.

**Figure 7:**
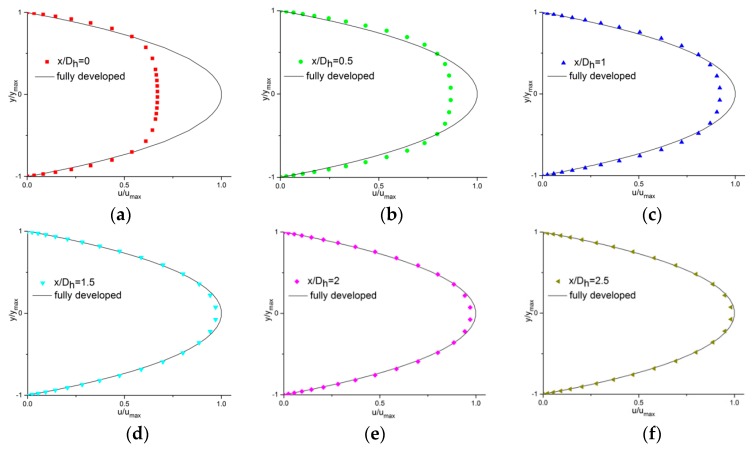
Velocity profiles at different locations along the axial length of the microchannel with $D_{h}=100\;\mu m$ (*Re* = 50). (**a**) $x/D_{h}=0$; (**b**) $x/D_{h}=0.5$; (**c**) $x/D_{h}=1$; (**d**) $x/D_{h}=1.5$; (**e**) $x/D_{h}=2$; (**f**) $x/D_{h}=2.5$.

Figure 7 presents the velocity profiles at different locations along the axial length of the microchannel with $D_{h}$ *=* 100 μm at *Re =* 50. The local velocity (*u*) was normalized with the fully developed axial velocity at the centerline ($u_{max}$) and the spanwise direction (*y*) was normalized with half of the microchannel width (w/2 = $y_{max}$). Theoretical fully developed velocity profiles gained from the Navier–Stokes equation in White et al.’s research [26] are shown in each plot with a solid line. 

As Figure 7 shows, the value of the $u_{c}⁄u_{max}$ (the local axial velocity at the centerline divided by the developed axial velocity at the centerline) at the plane of $x/D_{h}$ = 0 was about 0.65, and this value was 0.99 at the plane where $x/D_{h}$ = 2. In this case, the entrance length was less than $x/D_{h}$ = 2.

These results agree well with the physical mechanisms of the developing flow. It can be observed that the velocity of the fluid in contact with the walls reduced to zero as soon as the fluid entered the channel from the reservoir and the fluid adjacent to the wall was decelerated due to the viscosity. However, this effect diminished towards the central zone of the channel, causing the central line to have the maximum velocity. The velocity profile continued to transform along the axial length of the channel until it became parabolic and no further changes occurred, which is considered as fully developed.

**Figure 8:**
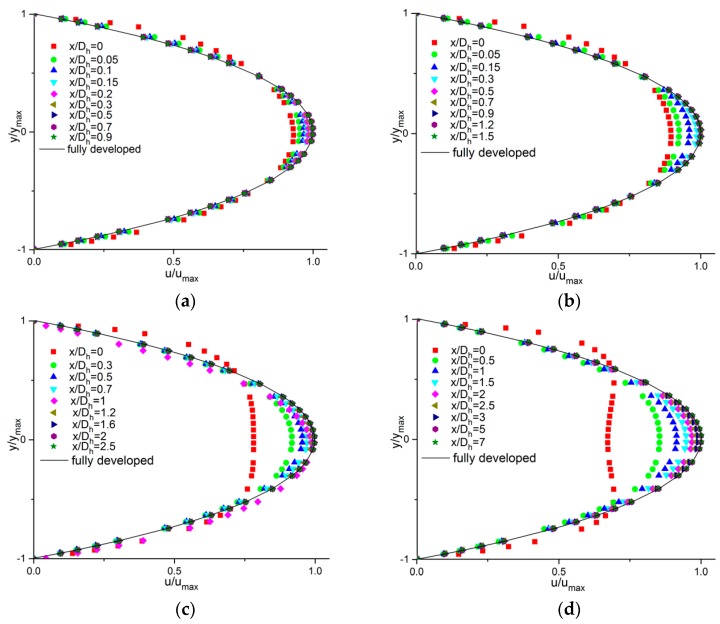
Developing velocity profiles along the axial length at different Reynolds numbers; $D_{h}=200\;\mu m.$ (**a**) *Re* = 0.5; (**b**) *Re* = 5; (**c**) *Re* = 20; (**d**) *Re* = 50.

The developing velocity profiles along the axial length at different Reynolds numbers in microchannels with 200 µm hydraulic diameter are shown in Figure 8. The values of $u/u_{max}$ at the entrance of the channel ($x/D_{h}$) reduced as the Reynolds numbers increased. The initial profiles were already parabolic and approximated the fully developed velocity profile when *Re* were lower. However, with increasing *Re*, a flatter inlet velocity profile could be observed at the entrance with a lower value of $u/u_{max}$, requiring a greater distance to achieve the fully developed velocity profile.

### 3.3. Influence of Roughness

Over the past half century, people studying laminar flow at conventional scale have obtained an accepted conclusion that there is no significant effect on laminar flow in channels when the relative roughness of the channel wall is less than 5% [27]. However, there is no recognized conclusion for microchannels. Hence, simulations were performed to determine the influence of roughness on the entrance effects in microchannels.

**Figure 9:**
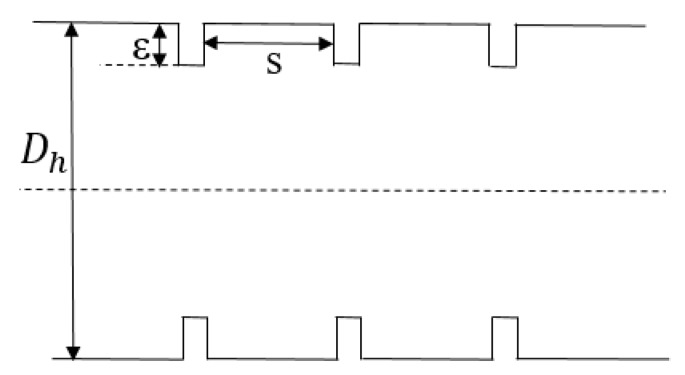
Local rough element distribution in the channel.

Rough elements were simulated by fins distributed along the length of the channel, and their local distribution is shown in Figure 9. The hydraulic diameter is described as $D_{h}$, the height of the roughness element as *ε*, and the distance between two adjacent rough elements as *s*. 

#### 3.3.1. Height of the Rough Elements

Simulations were performed in four different channels. The hydraulic diameters were all 200 µm ($D_{h}$ = 200 μm) and the distances between the adjacent rough elements were 50 µm (*s* = 50 µm). The working fluid was deionized water and the Reynolds number ranged from 0.5 to 100. Relative roughness (*r*) is defined as the height of rough element divided by hydraulic diameter (*r* = ε/$D_{h}$). The relative roughness of the channels was 0 (ε = 0), 1% (ε = 2 µm), 3% (ε = 6 µm), and 5% (ε = 10 µm), respectively.

**Figure 10:**
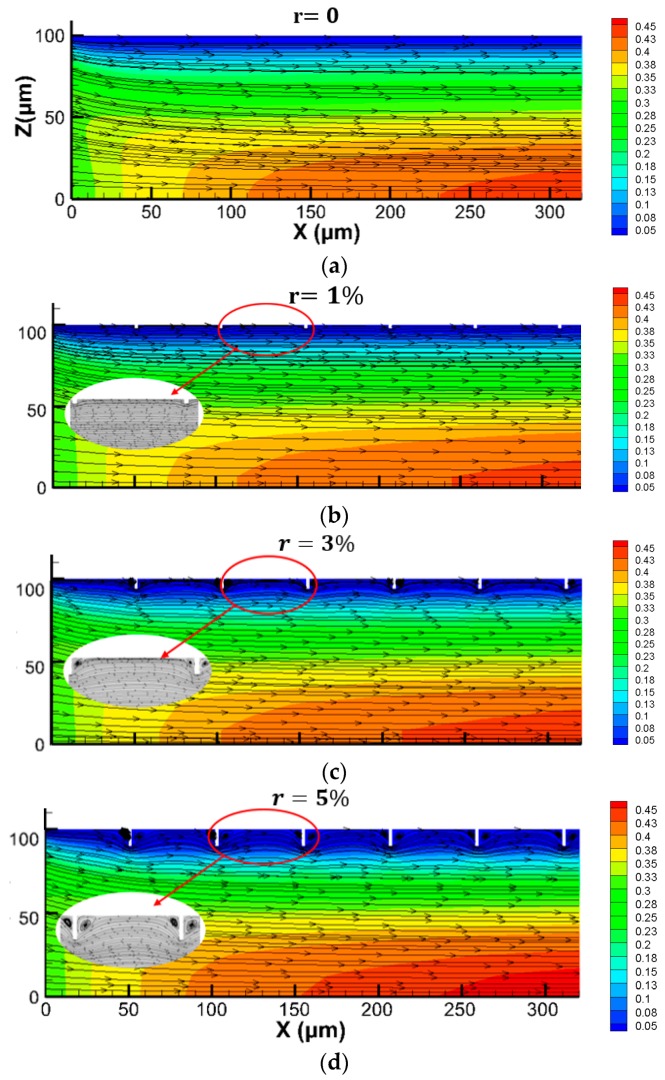
Velocity nephograms and streamlines of the microchannels with relative roughnesses r of 0%, 1%, 3%, and 5%. (**a**) r = 0; (**b**) r = 1%; (**c**) r = 3%; (**d**) r = 5%.

Figure 10 presents the velocity nephograms of the models with relative roughnesses of 0%, 1%, 3%, and 5%, respectively, when the Reynolds number was 50 in the plane *y =* 0. To make the flow conditions clearer, the streamlines of each channel are shown at the same time. As Figure 10 illustrates, the presence of the roughness elements significantly changed the flow state of the fluid by generating vortexes compared with the models without roughness elements. The vortexes became larger as the relative roughness increased.

According to the research [27], there is no significant effect on laminar flow in channels when the relative roughness of the channel wall is less than 5%. This conclusion has been widely accepted by scholars. The friction factor of the microchannel is given by $f=\frac{2\Delta pD_{h}}{\rho L{u_{m}}^{2}}$, where L and Δp are the length of the microchannel and pressure drop, respectively. Moddy’s chart indicates that the friction factor should satisfy f=64/Re in conventional-scale channels when the relative roughness is less than 5%.

**Figure 11:**
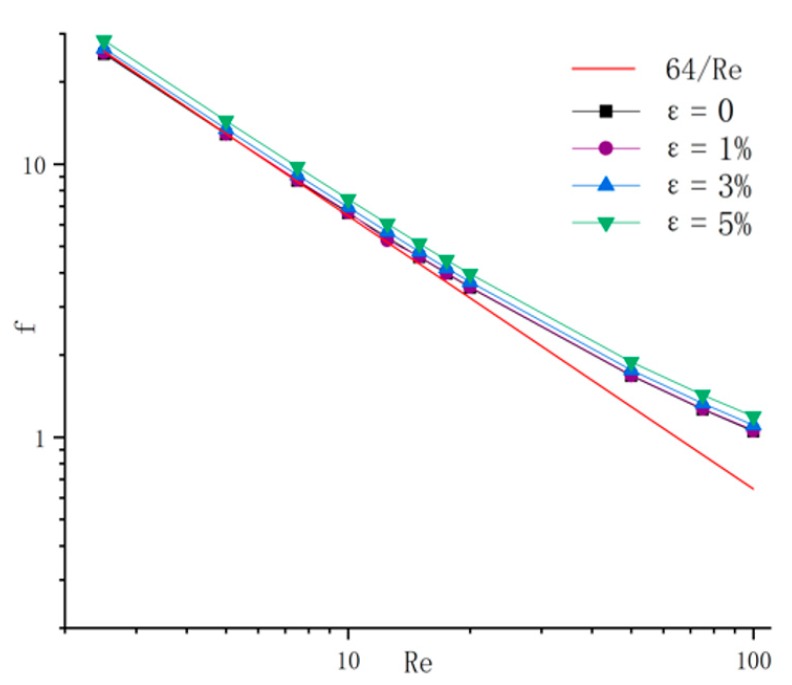
Friction factors *f* in microchannels with different relative roughnesses and the conventional scale conclusion that *f =* 64*/Re.*

The friction factors in microchannels with different relative roughnesses are shown in Figure 11. The theoretical values in conventional-scale channels are shown in a red solid line at the same time (64/Re). As shown in Figure 11, the friction factors in microchannels were higher than in conventional-scale channels, and the gap became more distinct for higher Reynolds numbers. Additionally, in contrast to the conventional scale, the roughness in microchannels led to increased friction factor, and the increased scope became more evident in the channels with higher relative roughness.

**Figure 12:**
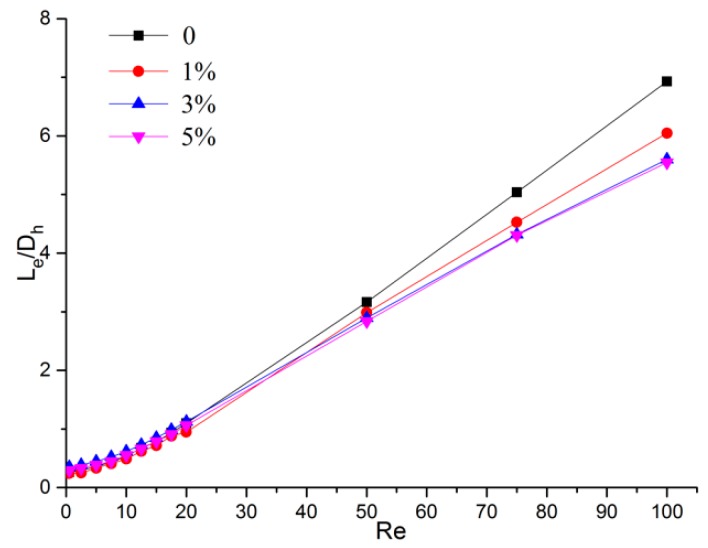
Dimensionless entrance region length versus the Reynolds number of microchannels with different relative roughness.

The dimensionless entrance region lengths versus the Reynolds numbers for microchannels with different relative roughnesses are shown in Figure 12. It can be observed that the existence of the rough elements obviously shortened the dimensionless entrance region, which is inconsistent with the conventional scale [27]. This became more evident as the relative roughness increased. It can also be observed that this distinction became more obvious as the Reynolds number increased. However, the values of $L_{e}/D_{h}$ were almost the same for models with 3% and 5%. Hence, it seems that there is a limit to the effect of shortening the entrance region by increasing the roughness height. 

#### 3.3.2. Spacing of Rough Elements

In order to determine how the distance of two adjacent rough elements (*s*) affects the dimensionless entrance region length, simulations were performed for *s =* 25, 50, 100, and 150 µm with 0.5 ≤ *Re* ≤ 100. The hydraulic diameters of both channels were 200 µm ($D_{h}$ = 200 μm) and the rough element heights were all 6 µm (ε = 6 µm). 

**Figure 13:**
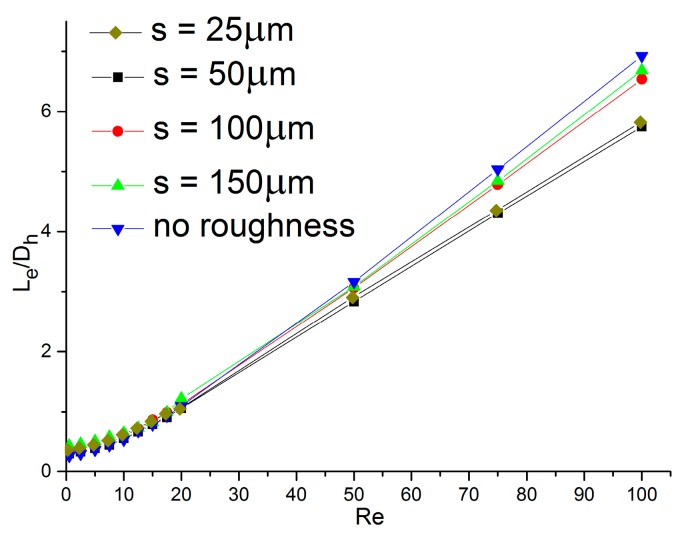
Dimensionless entrance region length versus *Re* for microchannels with different rough element distances.

The results obtained from the simulations are shown in Figure 13. For a given Reynolds number, the model without roughness had the longest entrance length and the channel with a distance of 50 µm had the shortest one. For *Re* = 5, the dimensionless entrance region length was 0.39 when *s =* 50 µm, and it was 0.42 when *s =* 100, and the relative discrepancy was 8.2%. However, when *Re* = 100, this discrepancy increased to 17.9%, with the values of dimensionless entrance length 5.55 and 6.54, respectively. Therefore, the distance of two adjacent rough elements had a significant effect the entrance effect. The following can be seen from the graphs: (1) Compared with the smooth channels, the rough ones had the shorter entrance lengths; (2) the entrance length decreased with decreasing distance and this became more notable with higher Reynolds numbers.

Note that the values of the model with *s =* 25 µm were a little higher than for that where *s =* 50 µm. This seems to contradict the above conclusion. However, according to Jia et al.’s research [28], when the distance between two rough elements is less than 30 µm, the fluid in microchannels may slip, increasing the entrance length.

#### 3.3.3. Symmetric Distribution

**Figure 14:**
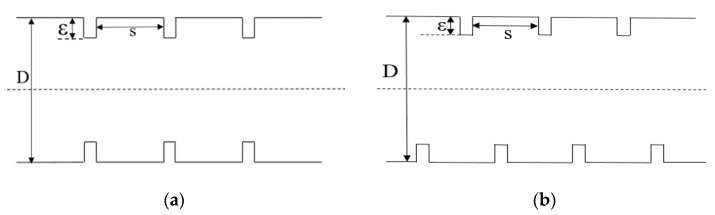
Structures of symmetrically and asymmetrically distributed rough elements. (**a**) Symmetric distribution; (**b**) Asymmetric distribution.

Simulations with different rough element distributions were performed for further study. As Figure 14 shows, the hydraulic diameter ($D_{h}$), height (ε), and rough element distance (s) of the two channels were exactly the same, as $D_{h}$ = 200 μm, ε = 2 μm, and *s* = 50 μm. Therefore, the only difference between the two models was in the distribution of their rough elements. In the first channel, as shown in (a), the rough elements were symmetrically distributed on the upper and lower surfaces, while in the second channel (b), the rough elements were staggered.

**Figure 15:**
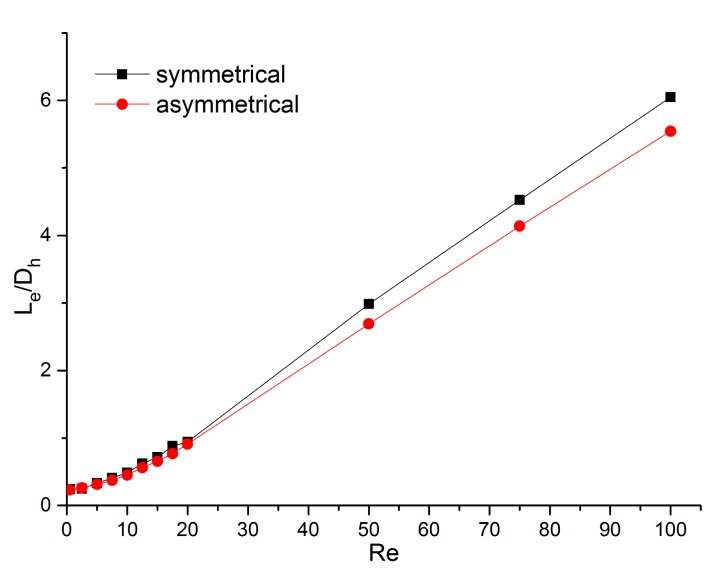
Dimensionless entrance region length versus *Re* in channels with different roughness distributions.

The dimensionless entrance region length versus the Reynolds number of the above two channels are shown in Figure 15. For *Re* = 0.5, the difference between the two channels was slight—0.242 for the channel with symmetrically distributed rough elements and 0.232 for the other channel, and the relative gap was only 4.3%. However, when the Reynolds number was increased to 100, the values became 6.05 and 5.54, respectively, and the relative discrepancy was 9.2%. This indicates that a staggered distribution of rough elements can decrease the dimensionless entrance region length and this became more obvious for higher Reynolds numbers. It can be extrapolated that other irregular distributions of rough elements may have the same impact.

## 4. Conclusions

Numerical simulations of fluid flow in square and rectangular microchannels were conducted in the laminar regime with Reynolds numbers ranging from 0.5 to 100. The hydraulic diameters of the microchannels were 50, 100, 150, and 200 μm with aspect ratios 1, 1.25, 1.5, 1.75, and 2. Microchannels with rough elements were also investigated. The main conclusions reached are as follows:

(1) The decisive factors of the dimensionless entrance region length in the rectangular microchannel were the Reynolds number and the aspect ratio. The dimensionless entrance region lengthened with increasing Reynolds number and decreasing aspect ratio. For a given Reynolds number, the dimensionless entrance region was longest in a square channel.
(2) Correlations of the dimensionless entrance length as a function of Reynolds number are proposed in rectangular channels of different aspect ratios (1, 1.25, 1.5, 1.75, 2). These correlations can be extrapolated to other laminar flows with higher Reynolds numbers to predict the entrance length in microchannels.
(3) The existence of rough elements obviously shortened the entrance region length, and this became more notable as the relative roughness and the Reynolds number increased. A similar effect could be obtained by shortening the distance between the rough elements. Compared with symmetrically distributed the rough elements, asymmetric distribution decreased the entrance length, which can be extrapolated to other irregular distribution forms.

## Figures and Tables

**Table 1:** Coefficients in Equations (1)–(3).

Correlations	$C_{1}$	$C_{2}$	$C_{3}$
Atkinson et al. [13]			
Tube	0.590	0.056	–
Parallel plates	0.625	0.044	–
Chen et al. [14]			
Tube	0.600	0.035	0.056
Parallel plates	0.630	0.035	0.044
Schlichting et al. [15]	0.060	–	–

**Table 2:** Conclusions of some previous research on the entrance region in microchannels.

Reference	Year	Conclusions
Lee and Kim et al. [20]	2003	The entrance length of the microscale devices is much shorter than that of macroscale channels
Ahmad and Hassan et al. [21]	2010	$\frac{L_{e}}{D_{h}}=\frac{0.6}{0.14Re+1}+0.0752Re$
Hao et al. [22]	2005	$\frac{L_{e}}{D_{h}}=0.08-0.09Re$
Renksizbulut and Niazmand et al. [23]	2006	$\frac{L_{e}}{D_{h}}=\left[0.085Re+\frac{0.8}{Re^{0.3}}\right]\left[{\left(\frac{90{}^{\circ}}{\varphi }\right)}^{0.6}\right]\left[{\left(1+\alpha \right)}^{-0.24}\right]$
Galivis and Yarusevych et al. [24]	2012	$\frac{L_{e}}{D_{h}}=\frac{0.74}{0.09Re+1}+0.0889Re$

**Table 3:** Grid independence analysis at *Re* = 50.

Case	Elements	Nodes	r	Δp, Pa	Δp Error, Δ
Grid1	529,880	484,288	–	2219.37	–
Grid2	953,374	885,076	1.8	2246.68	1.2%
Grid3	1,608,830	1,511,300	1.7	2275.61	1.3%
Grid4	2,163,943	2,042,460	1.3	2316.55	1.8%
Grid5	3,170,380	3,018,256	1.5	2328.69	0.5%

**Table 4:** Parameters of the five microchannels.

Model	Width (µm)	Height (µm)	Aspect ratio (*w/h*)	$D_{h}$ (µm)
1	200	200	1	200
2	225	180	1.25	200
3	250	167	1.5	200
4	275	157	1.75	200
5	300	150	2	200

**Table 5:** Coefficients of the proposed correlations.

Aspect Ratio (w/h)	$C_{1}$	$C_{2}$	$C_{3}$	$C_{4}$	$C_{5}$
1	0.263	0.267	0.0471	0.0707	−0.29
1.25	0.286	0.050	0.0310	0.0624	−0.349
1.5	0.338	0.070	0.0330	0.052	−0.18
1.75	0.370	0.054	0.0291	0.0419	−0.055
2	0.421	0.061	0.0265	0.0302	0.1631

## Nomenclature

$D_{h}$	hydraulic diameter, m
Re	Reynolds number
L	channel length, m
w	channel width, m
h	channel height, m
w/h	channel aspect ratio (width/height)
$L_{e}$	length of the entrance region, m
$L_{e}/D_{h}$	dimensionless entrance region length
u	velocity, m/s
$u_{max}$	maximum velocity, m/s
$y_{max}$	channel half height, m
$u_{FD}$	theoretical fully developed velocity, m/s
ρ	density, $\mathrm{kg}/m^{3}$
µ	dynamic viscosity, Pa·s
ε	rough element height, m
*s*	adjacent rough elements distance, m
